# Extracting candidate motif patterns for multiple RNA modifications using direct RNA sequencing

**DOI:** 10.1186/s12859-026-06724-5

**Published:** 2026-10-23

**Authors:** Nicolò Alagna, Johannes Miedema, Yahia Basiony, Susanne Gerber

**Affiliations:** 1 Institute of Human Genetics, University Medical Center Mainz, Mainz, Germany; 2 Institute for Quantitative and Computational Biosciences (IQCB), Mainz, Germany

**Keywords:** RNA modifications, Epitranscriptomics, Nanopore direct RNA sequencing, Sequence motif discovery, Exponential population analysis, Bioinformatics

## Abstract

RNA modifications have emerged as key regulators of virtually all aspects of RNA biology, driving growing interest in computational approaches for their transcriptome-wide detection. For some RNA modifications, the search space can be reduced by exploiting characteristic sequence motifs, as exemplified by the canonical DRACH motif associated with m⁶A. However, comparable sequence preferences remain largely unknown for most other RNA modifications, making systematic motif discovery considerably more challenging. Here, we applied exponential population analysis to direct RNA sequencing data from HEK293T cells and peripheral human blood to identify candidate motif patterns for pseudouridine (Ψ), inosine (I), and 5-methylcytosine (m⁵C). Our results demonstrate that exponential population analysis provides a systematic framework for identifying candidate motif patterns from nanopore-derived RNA modification predictions, thereby prioritizing sequence contexts for future biological validation.

## Introduction

Chemical modifications of the RNA can affect virtually every stage of the cellular life cycle [1], including molecular structure, protein interactions, and cellular metabolism [2–5]. Abnormal accumulation or depletion of RNA modifications at sites where they would normally occur can profoundly influence cell fate, leading to cellular dysfunction and disease [6–9]. To date, more than 170 different types of RNA modification have been identified, at least 50 of which occur in humans, highlighting the complexity of the epitranscriptomic landscape [10]. Among the most intensively studied RNA modifications are pseudouridine (Ψ) [3, 11, 12], inosine (I) [13, 14], N6-methyladenosine (m⁶A) [15–19], and 5-methylcytosine (m⁵C) [20, 21]. Dysregulation of these and other RNA modifications has been implicated in a broad spectrum of human diseases, including neurodegenerative disorders, immunological deficiencies, and multiple forms of cancer [6–9, 22].

Several methods have been developed to study the epitranscriptomic landscape. Examples include MeRIP-Seq [23] m6ACE-Seq [24], miCLIP [25], Pseudo-seq [26], and GLORI [27]. Although these approaches have led to major advances, short-read sequencing technologies often struggle to comprehensively detect the full diversity of RNA modifications [2, 28, 29]. More recently, Oxford Nanopore Technologies introduced direct RNA sequencing (DRS), a long-read sequencing approach that is transforming epitranscriptomics by enabling the direct detection of RNA modifications at single-nucleotide resolution [30–37].

These approaches also enable the identification of so-called sequence motifs, i.e., short nucleotide sequences in which specific RNA modifications occur with particularly high frequency. The best-characterized example is the m⁶A DRACH motif (5′-DRACH-3′, where A denotes the methylatable adenosine, D represents A, G, or U, R represents A or G, and H represents A, C, or U). This evolutionarily conserved motif is widely distributed across cellular and viral transcriptomes, and has been shown to encompass approximately 85% of all currently known m⁶A sites [38, 39, 40]. However, analogous motif patterns have not yet been identified for most other RNA modifications. This is largely due to the limited and uneven representation of experimentally supported modification sites in current databases [39–46], particularly compared with the extensive annotation available for m⁶A.

In this study, we analyzed direct RNA sequencing (DRS) data from HEK293T cells and peripheral human blood to identify candidate motif patterns for m⁶A, inosine (I), pseudouridine (Ψ), and m⁵C. Specifically, we combined Dorado for basecalling, Modkit for modification site extraction, and exponential population analysis [41, 42] to characterize sequence motifs associated with these RNA modifications. As a proof of concept, we first analyzed all predicted m⁶A sites in both sample types using population analysis to reconstruct the well-established DRACH motif.

As expected, and in agreement with previous studies, the analysis resolved two distinct populations (or motif families): (i) a high-frequency population containing the canonical DRACH motifs and (ii) a low-frequency population comprising additional m⁶A sites located outside the DRACH sequence context. Having demonstrated that our approach can reconstruct the canonical DRACH motif, we subsequently applied the same analytical framework to investigate the sequence context of three additional RNA modifications (inosine, pseudouridine, and m⁵C) and to identify candidate motif patterns for each.

## Results

HEK293T cells and peripheral human blood samples were analyzed by direct RNA sequencing (DRS) to identify 5-mer sequence motifs associated with four RNA modifications (m⁶A, Ψ, I, and m⁵C). Specifically, two HEK293T replicates and three peripheral human blood replicates were processed using Dorado for basecalling and Modkit for modification site extraction (see Methods for details). The resulting modification sites, together with their surrounding nucleotide sequences, were subsequently used to analyze the corresponding 5-mer sequence contexts.

### m^6^A population analysis on collected 5-mers

**Fig. 1:**
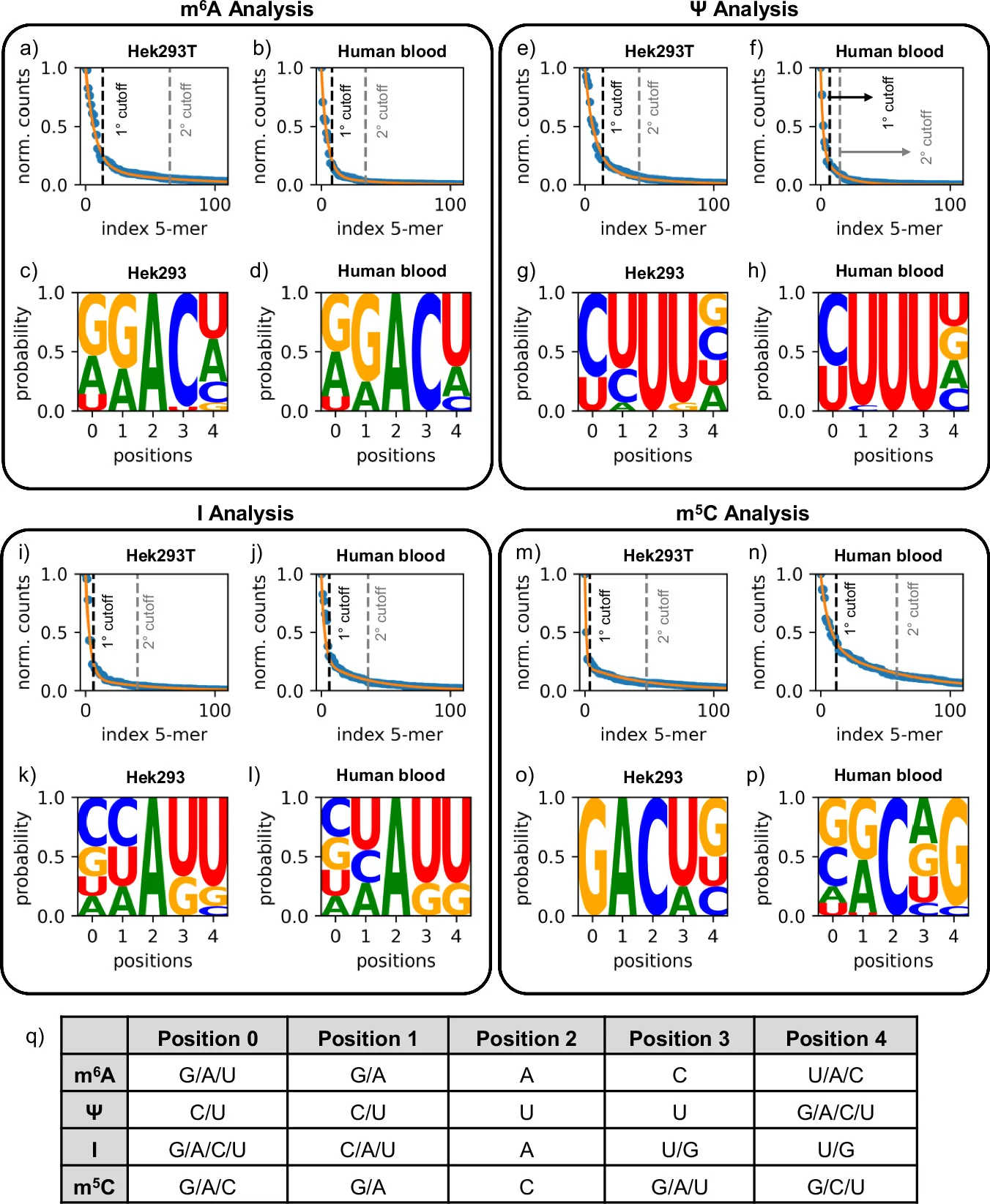
Normalized count*s* over indexed 5-mer (blue circles) with relative population fitting (orange line) for m^6^A (**a**,**b**), Ψ, (**e**,**f**), I (**i**,**j**) and m5C (**m**,**n**) for Hek293 and human peripherical blood samples, respectively. Selecting the first population (underlined by each motif before the vertical black dashed line) a logo plot was calculated for m^6^A (**c**,**d**), Ψ, (**g**,**h**), I (**k**,**l**) and m5C (**o**,**p**) for both cell lines. Clear motif paths can be observed, which can be reproduced in both cell lines analyzed here. Vertical grey dashed lines show the cutoff index including the second population. **q** Combinations of 5-mer motifs for m^6^A, I, Ψ and m^5^C. Each position (zero-indexed) shows the possible nucleotide that can be used to obtain all 5-mer combinations of the motifs appearing with high frequency

We began by analyzing the datasets to determine how frequently each modified 5-mer was detected following m⁶A basecalling with Dorado. The identified 5-mers were subsequently ranked in descending order according to their frequency. Figures 1a and 1b show the normalized abundance of each detected 5-mer, with each blue circle representing a single motif. The resulting distribution was fitted using a double-exponential function (orange line, Figs. 1a and 1b), revealing the presence of two distinct populations of m⁶A-associated 5-mers (see Methods). The first population comprises a relatively small number of highly abundant motifs, whereas the second consists of motifs occurring at low to very low frequencies.

All replicates from each sample type (HEK293T and peripheral human blood) were fitted simultaneously using the same double-exponential model. The corresponding fit parameters are summarized in Supplementary Tables SI1–SI4. Comparison of the fitted amplitudes (A₁ and A₂) showed that the first population exhibited amplitudes approximately five- to tenfold higher than the second population in both sample types.

For example, in the first HEK293T replicate, the fitted amplitudes were A₁ = 22,800 and A₂ = 3,755, whereas the corresponding values for human blood were A₁ = 22,246 and A₂ = 2,741 (Supplementary Table SI1). Similar ratios were observed across all replicates.

The double-exponential fit additionally yields the characteristic decay constants (λ₁ and λ₂), which were used to determine the cutoff separating the two populations (see Methods). Applying the cutoff at 2 × λ₁ (vertical dashed line in Figs. 1a and 1b), sequence logos generated from all motifs up to this threshold accurately reconstructed the canonical DRACH motif in both HEK293T and human blood samples (Figs. 1c and 1d). Minor differences between the two sample types were observed only at positions 3 and 4 and were considered noise, as their average nucleotide contribution remained below the predefined 6% threshold (see Methods, Eq. 5).

For clarity, the sequence logo shows the probability of each nucleotide at every position of the zero-indexed 5-mer. The modified nucleotide is located at the central position (pos 2) and is represented by its corresponding canonical base. In contrast, the sequence logo generated from the second population exhibits a largely random pattern that cannot be associated with the canonical DRACH motif (Supplementary Figure S1). These results demonstrate that the proposed population analysis accurately reconstructs the expected DRACH motif by fitting a double-exponential function to the ranked distribution of modified 5-mer abundances.

### Extending population analysis on Ψ, I and m^5^C

After validating the approach by successfully reconstructing the DRACH motif from predicted m⁶A sites using population analysis, we applied the same workflow to the other RNA modifications and analyzed all detected positions of Ψ, I, and m⁵C. Specifically, for each RNA modification, we performed double-exponential fitting, calculated the cutoff index (2 × λ₁), generated the sequence logo for the first population, filtered low-frequency noise (see Methods, Eq. 5), and extracted modification-specific candidate motif patterns.

Starting with Ψ, a clear double-exponential fit of the motif abundance distribution was observed for both sample types (Figs. 1e and 1f). The sequence logos of the first population (Figs. 1g and 1h) revealed a highly consistent motif pattern across HEK293T and peripheral human blood samples, which can be summarized as pos0 = C/U, pos1 = C/U, pos2 = U, pos3 = U, and pos4 = N, where N denotes any nucleotide (A/C/G/U). The only noticeable difference between the two sample types was the minor contribution of A (5–6%) and G (5–6%) at positions pos1 and pos3 in the HEK293T samples (Fig. 1g) compared with the human blood samples. Whether this observation reflects biological variation, statistical noise, or a minor contribution from the second population cannot be determined at present.

Similar to Ψ, the inosine sequence logos (Figs. 1k and 1l) also revealed a distinct motif pattern for the high-frequency population (Figs. 1i and 1j). The extracted motif combination was defined as pos0 = N, pos1 = C/A/U, pos2 = A, pos3 = U/G, and pos4 = U/G, and was consistent across all replicates. The only difference observed between the two sample types was the additional contribution of C (8–9%) at position pos4 in the HEK293T samples (Fig. 1k). Given its relatively high abundance, this observation may indicate a cell-type-specific sequence preference in HEK293T compared with peripheral human blood.

In contrast, m⁵C exhibited a more complex pattern than the other RNA modifications investigated. The population distributions differed considerably between the two sample types. Whereas a distinct motif pattern was observed in the HEK293T samples, m⁵C sites detected in the human blood replicates appeared to be distributed more randomly (Figs. 1m and 1n). This observation is consistent with the stronger contribution of the second population in human blood (see A₁ and A₂ in Supplementary Table SI4), where the amplitude ratio (A₂/A₁ ≈ 65%) was substantially higher than that observed for the HEK293T samples (A₂/A₁ ≈ 25%). Although this interpretation remains speculative, we hypothesize that these differences are unlikely to reflect tissue-specific biological variation, but rather result from the relatively high false-positive rate reported for nanopore-based m⁵C detection in native (non-synthetic) RNA samples.

Despite these observations, we attempted to extract a candidate motif pattern for m⁵C by identifying the minimal motif combination that best represented both sample types. The resulting motif combination was defined as pos0 = G/A/C/U, pos1 = G/A, pos2 = C, pos3 = A/G/U, and pos4 = G/C/U. The motif patterns identified for m⁶A, Ψ, I, and m⁵C are summarized in Fig. 1q.

To provide a quantitative overview of the modification calls obtained in the two experimental systems, we summarized the total number of sites detected by Dorado/Modkit and the number assigned to the high-frequency population for each modification type (Supplementary Table SI6). Across the two HEK293T replicate datasets, we obtained 1,001,294 m⁶A, 515,602 Ψ, 65,891 inosine, and 171,832 m⁵C site calls. Of these, 465,545 m⁶A (46.5%), 293,922 Ψ (57.0%), 26,919 inosine (40.9%), and 31,925 m⁵C calls (18.6%) were assigned to the high-frequency population. Across the three peripheral blood replicate datasets, we obtained 353,528 m⁶A, 94,970 Ψ, 75,004 inosine, and 165,992 m⁵C site calls, of which 203,940 m⁶A (57.7%), 57,304 Ψ (60.3%), 23,516 inosine (31.4%), and 47,923 m⁵C calls (28.9%) belonged to the high-frequency population. These values provide a descriptive overview of modification calling in the two systems but should not be interpreted as biological differences in modification abundance, because the datasets differ in sample composition, sequencing depth, and transcript representation.

To assess whether the observed motif patterns could reflect sequence-dependent biases of nanopore modification calling rather than genuine biological preferences, we followed three complementary strategies. First, we investigated whether similar motif patterns could be identified by comparing the predicted modification sites from our study with modification–motif combinations reported in published datasets generated using orthogonal methods. Second, we applied our population analysis approach to these published datasets to determine whether the high-frequency motif patterns identified in our study could be reproduced. Third, we analyzed the IVT data generated for our human blood samples as negative controls to assess potential false-positive predictions.

We first compared the motif patterns identified in our analysis with published data from the m5C-Atlas, m6A-Atlas V2, Ψ-Atlas, and RMBase V3 databases [43–46]. The motif overlap analysis showed that the motif patterns identified in this study (Fig. 1q) covered 84%, 12.5%, 29%, and 15.9% of all currently documented positions for m⁶A, Ψ, I, and m⁵C, respectively.

It should be noted, however, that the databases for Ψ, I, and m⁵C contain substantially fewer annotated positions than the m⁶A database, which currently comprises approximately 900,000 annotated sites [43, 44]. Specifically, the Ψ, I, and m⁵C databases contain only approximately 0.6% [6k], 11% (100k), and 5.1% [46k], respectively, of the annotated positions currently available for m⁶A.

The relatively small number of annotated positions may result in high- and low-frequency motifs being represented in similar proportions within these databases, thereby reducing the statistical power of the analysis and contributing to the relatively low overlap between the motif patterns identified in this study and the corresponding 5-mer patterns reported for Ψ, I, and m⁵C.

Indeed, when a larger fraction of the second population was included in the motif analysis (grey vertical dashed line in Fig. 1; second cutoff = λ₂, Supplementary Tables SI1–SI4), the overlap increased to 98%, 89%, 92%, and 66% for m⁶A, Ψ, I, and m⁵C, respectively. This observation further underscores the importance of generating larger transcriptome-wide datasets of validated RNA modification sites to improve the statistical identification of overrepresented and underrepresented sequence motifs.

Next, we applied our method to the documented 5-mer motifs of RNA modification sites obtained from the public databases. This analysis showed that, with the exception of m⁶A, no dominant motif population could be extracted from the database-derived datasets. Instead, the resulting sequence logos resembled those obtained for the second (low-frequency) m⁶A population identified by Dorado (Supplementary Figure S1).

**Fig. 2:**
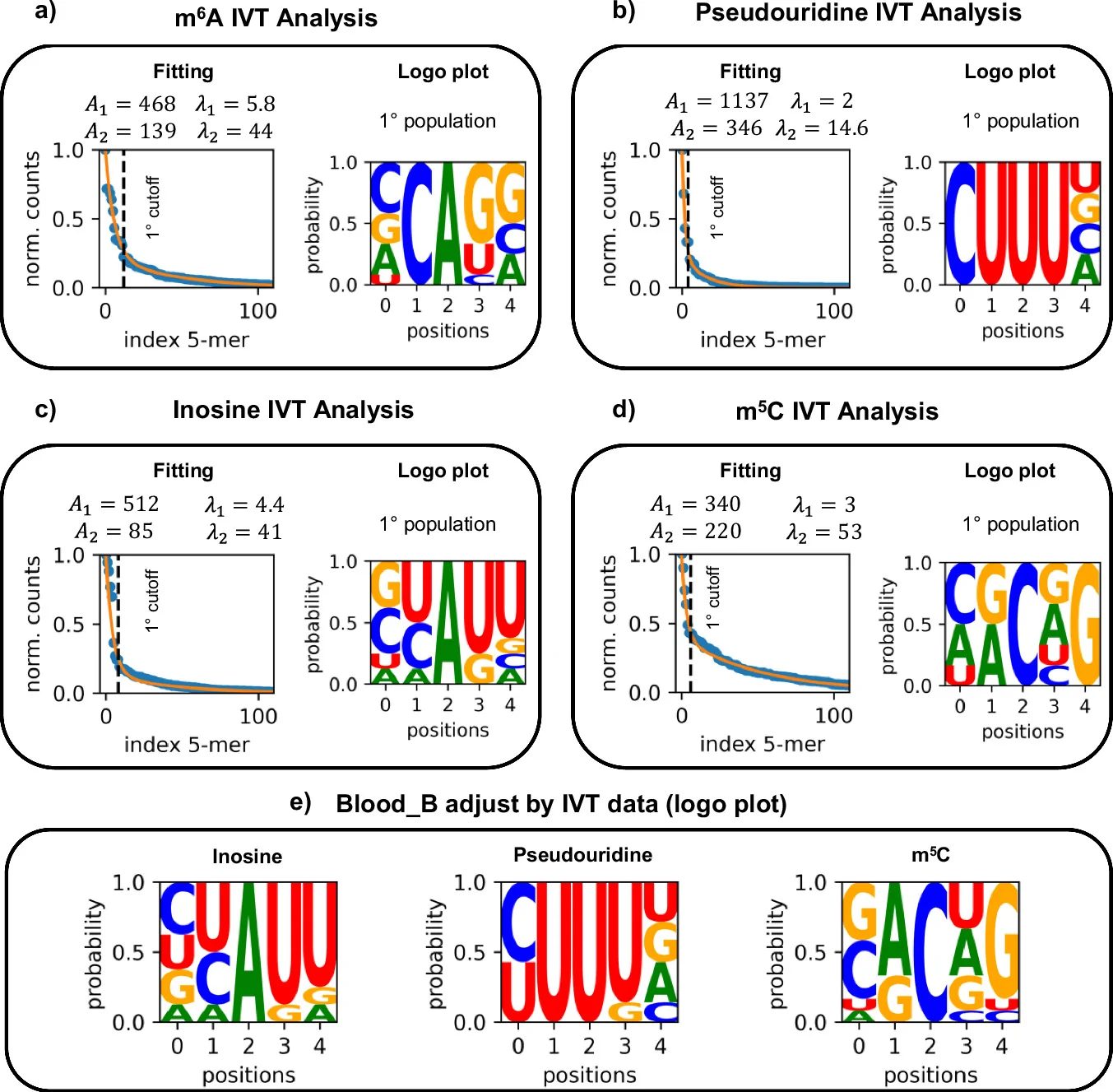
Population analysis performed for m^6^A (**a**), Ψ(**b**), I(**c**) and m^5^C (**d**) IVT data for the human blood samples. Each square contains Normalized counts over indexed 5-mer (blue circles) with relative population fitting (orange line) and 1^st^ cutoff (vertical dashed line); additionally, the 1^st^ population logo plots are shown for the IVT analysis of each RNA modification type. **e** logo plot for the Blood_B population analysis after IVT correction. Logo plots as practically not affected by IVT

Finally, we applied our population analysis to the matched *in vitro*-transcribed (IVT) controls of the human blood samples to assess whether systematic modification calls in unmodified RNA could bias the inferred motif patterns (Fig. 2a–d). For Ψ, inosine, and m⁵C, the fitted amplitude of the high-frequency population (A₁) in the IVT controls corresponded to approximately 10–20% of that observed in the respective native RNA samples, whereas for m⁶A it was below 1% (compare Fig. 2a–d with Supplementary Tables SI1–SI4). We therefore corrected the native blood data by subtracting the IVT-derived count of each 5-mer from the corresponding count in the native sample, refitted the resulting distributions, and regenerated the sequence logos. The principal motif patterns for Ψ, inosine, and m⁵C were preserved after IVT correction, with only minor differences at position 4 for inosine and position 1 for Ψ (compare Fig. 2e with Fig. 1h, k, and p). This indicates that the inferred population-level motif patterns are not solely driven by systematic modification calls in the IVT controls. Comparable modification calls in independently generated human IVT data have recently been reported by Tzadikario et al. [47].

Using Eq. 4, we estimated the IVT-derived false-positive contribution to the high-frequency population to be 2%, 3%, 10.7%, and 36% for m⁶A, Ψ, inosine, and m⁵C, respectively. The substantially higher value for m⁵C is consistent with the greater variability of its sequence logos between HEK293T and blood and supports a more cautious interpretation of the corresponding candidate motif. To further benchmark modification calling in systems with independently established modification sites, we analysed previously published synthetic oligonucleotides containing m⁶A, Ψ, or inosine [48] and published human ribosomal RNA nanopore data [49], using the experimentally mapped Ψ sites reported by Taoka et al. as the reference set [50] (Supplementary Information, Section SI2). Dorado recovered the expected modification site in 26 of the 30 synthetic oligonucleotides (86.7%). In the rRNA dataset, 93 of 104 known Ψ sites were recovered, corresponding to a recall of 89.4%, while 93 of the 115 sites predicted by Dorado corresponded to known Ψ positions, yielding a precision of 80.9%. In densely pseudouridylated regions, most known sites remained detectable, although their predicted modification frequencies tended to be underestimated. Thus, neighbouring Ψ sites did not generally prevent qualitative site recovery in this reference dataset but affected quantitative estimates. Because this analysis was restricted to pseudouridylation in rRNA, modification-specific effects of neighbouring modifications on m⁶A, inosine, and m⁵C calling cannot be excluded.

These findings are consistent with previous reports that nanopore-based modification calling can generate false-positive and false-negative predictions and can over- or underestimate modification frequencies [18, 19, 48, 51]. Our population-based approach reduces the influence of low-frequency erroneous calls by restricting motif extraction to the high-frequency population and excluding low-contribution nucleotides from the resulting motif representation. Nevertheless, the identified sequence preferences should be regarded as candidate motif patterns rather than independently validated biological motifs. Transcriptome-wide reference datasets generated using orthogonal methods remain limited, particularly for Ψ, inosine, and m⁵C, and additional orthogonal datasets will therefore be required to validate and refine the candidate patterns identified here.

## Discussion

The 5-mer population analysis successfully separated canonical m⁶A DRACH motifs from non-DRACH motifs in both HEK293T cells and peripheral human blood DRS samples (Fig. 1a–d), consistent with the underlying population-based framework [41, 42]. The m⁶A analysis further showed that 46.5% of the detected HEK293T m⁶A calls and 57.7% of the blood-derived m⁶A calls were assigned to the high-frequency population containing the canonical DRACH motif family. In parallel, the same analysis was performed on the corresponding in vitro transcription (IVT) datasets. The low contribution of the first IVT population (compare A₁ in Fig. 2a with A₁ in Supplementary Table SI1) indicates that systematic false-positive predictions contribute only minimally to the observed motif patterns, arguing against a substantial bias in the analysis.

These results demonstrate that population analysis can separate distinct RNA motif profiles, such as canonical DRACH and non-DRACH motifs, and can be extended to investigate candidate motif patterns associated with other RNA modifications. After demonstrating that the approach could reconstruct the canonical m⁶A DRACH motif, we applied the same analytical workflow to Ψ, inosine, and m⁵C (Fig. 1e–p). The analysis revealed consistent candidate motif patterns for Ψ and inosine, whereas the pattern inferred for m⁵C was less consistent across the two sample types and should therefore be considered preliminary (Fig. 1q and the corresponding sequence logos in Fig. 1).

We additionally analysed the corresponding IVT datasets for Ψ, inosine, and m⁵C. Comparison of the IVT-corrected sequence logos with those derived from native RNA showed that the principal motif patterns for Ψ and inosine were largely preserved, indicating that systematic modification calls in the IVT controls had only a limited effect on these population-level patterns (compare Fig. 2e with the corresponding sequence logos in Fig. 1). In contrast, the estimated IVT-derived false-positive contribution to the high-frequency m⁵C population was approximately 36%, supporting a more cautious interpretation of the candidate m⁵C motif.

The high IVT-derived false-positive contribution estimated for m⁵C highlights the increased level of noise in the corresponding modification calls and limits the reliability of the inferred motif patterns. Consistent with this observation, the m⁵C sequence logos differed substantially more between HEK293T and peripheral human blood than those obtained for m⁶A, Ψ, and inosine (Fig. 1). Although our population analysis identified a candidate m⁵C motif pattern, this pattern should therefore be considered preliminary and requires confirmation using additional datasets and orthogonal experimental approaches. The candidate patterns identified for Ψ and inosine were more consistent across the two sample types; however, their overlap with currently available reference databases remained relatively low. Specifically, the candidate motifs covered 12.5%, 29%, and 15.9% of the documented Ψ, inosine, and m⁵C positions, respectively. These findings emphasize the need for larger transcriptome-wide reference datasets with independently validated modification sites to distinguish genuine sequence preferences from caller-specific biases and to validate or refine the candidate motif patterns identified here.

## Methods

### Extraction of bed files and motifs count

Direct RNA sequencing libraries for the HEK293T and human blood samples were prepared using the SQK-RNA004 kit according to the manufacturer’s protocol. Raw signal data were stored in POD5 format and basecalled using Dorado v1.1.2 with the super-accuracy model. Reads were aligned to the human hg38 reference genome, and aligned BAM files were generated. Modification calls were extracted using Modkit and the all-context model, with --filter-threshold 0.8 and --mod-thresholds 0.8 for m⁶A, Ψ, and inosine and --mod-thresholds 0.9 for m⁵C. Positions with a coverage below 30 or a predicted modification frequency below 10% were excluded. A custom Python script was then used to determine the abundance of each modified 5-mer. The resulting table contained the detected 5-mers and their absolute counts and was sorted in descending order of abundance.

### Population analysis

To distinguish highly represented motif patterns from motifs occurring at low frequencies, we fitted the ranked 5-mer count distributions using a double-exponential model. The model describes the observed distribution as the sum of two exponential components, representing a high-frequency population and a low-frequency population:

1 $$f\left(x\right)={A}_{1}* {e}^{-\frac{x}{{{\rm{\lambda }}}_{1}}}+\,{A}_{2}* {e}^{-\frac{x}{{{\rm{\lambda }}}_{2}}}\,$$

Here, *x* denotes the rank of a 5-mer in the abundance-sorted distribution. *A*₁ and *A*₂ represent the fitted amplitudes of the high- and low-frequency populations, respectively, while λ₁ and λ₂ are their corresponding decay constants. Each decay constant specifies the rank interval over which the contribution of the respective population decreases to 1/*e* of its initial amplitude.

To define a reproducible cutoff between the two populations, we calculated the signal ratio (*SR*) between the fitted contributions of the high- and low-frequency components:

2 $${S}_{R}=\frac{{Signa}{l}_{{family}1}(t=n* {\lambda }_{1})}{{Signa}{l}_{{family}2}(t=n* {\lambda }_{1})}=\frac{{A}_{1}* {e}^{-\frac{n* {\lambda }_{1}}{{{\rm{\lambda }}}_{1}}}}{{A}_{2}* {e}^{-\frac{n* {\lambda }_{1}}{{{\rm{\lambda }}}_{2}}}}=\frac{{A}_{1}}{{A}_{2}}{e}^{(-n+n* \frac{{\lambda }_{1}}{{\lambda }_{2}})}$$

The signal ratio was evaluated at *x* = *n* × λ₁ for different values of *n*. An *SR* of 1 indicates that the two fitted populations contribute equally to the total signal; at lower ranks, the high-frequency population dominates, whereas at higher ranks, the low-frequency population becomes predominant. Across all analysed datasets, the median *SR* was approximately 1 at *n* = 2 (Supplementary Table SI5a). We therefore defined the cutoff between the two populations as *x* = 2 × λ₁ and applied this criterion consistently to all RNA modification types and sample groups. A minimum value of λ₁ = 2 was required to ensure a sufficiently defined high-frequency population for subsequent motif extraction.

The contribution of each fitted population to the total signal was estimated by integrating the corresponding exponential component. For example, the contribution of the high-frequency population up to the defined cutoff was calculated as follows:

3 $${Contribution}={\int }_{0}^{\infty }{{{A}_{1}}^{e}}^{-\frac{x}{{\lambda }_{1}}}{dx}=\frac{{A}_{1}}{{\lambda }_{1}}$$

Matched in vitro-transcribed (IVT) controls were used to estimate the contribution of systematic modification calls in nominally unmodified RNA. For a selected population, the IVT-derived contribution was calculated by dividing the integrated signal obtained from the IVT control by the corresponding integrated signal obtained from the native RNA sample. For the high-frequency population, this estimate was calculated as follows:

4 $${False}\,{positive}\,{fraction}=\frac{{\int }_{0}^{\infty }{A}_{1{\rm{\_}}{IVT}}{e}^{-\frac{x}{{{\rm{\lambda }}}_{1{\rm{\_}}{IVT}}}}{dx}}{{\int }_{0}^{\infty }{A}_{1}{e}^{-\frac{x}{{{\rm{\lambda }}}_{1}}}{dx}}=\,\frac{{A}_{1{\rm{\_}}{IVT}}* {\lambda }_{1}}{{A}_{1}* {\lambda }_{1{\rm{\_}}{IVT}}}$$

The resulting value represents the estimated IVT-derived contribution to the high-frequency population and should not be interpreted as a general site-level false-positive rate of Dorado.

### Extraction of motif patterns from the sequence logos

Following population analysis, the 5-mers assigned to the high-frequency population were used to generate sequence logos showing the relative contribution of each nucleotide at each position surrounding the predicted modification site. To derive concise and reproducible motif representations, nucleotides with very low contributions, which may reflect noise or residual contributions from the second population, were excluded. A nucleotide was included in the candidate motif when its average contribution across the HEK293T and blood datasets was at least 6% at the respective position, as defined in Eq. 5:

5 $$\frac{{N}_{{human}}^{i}+{N}_{{HEK}293T}^{i}}{2}\,\ge 6 \% $$

Here, *N* denotes one of the four nucleotides (A, C, G, or U), and *i* denotes its position within the zero-indexed 5-mer (positions 0–4). The predefined 6% cutoff was applied consistently across all RNA modification types to remove low-contribution nucleotides and generate simplified representations of the dominant sequence patterns.

## Supplementary information

Supplementary Information

## Data Availability

Availability of data and materials Sequencing data was obtained from a previous study [18], which used SQK-RNA004 kits (Oxford Nanopore Technologies) for libraries preparation. Pod5 files for the HEK293T samples obtained from the European Nucleotide Archive (ENA) under the accession number PRJEB74238. Pod5 files for all blood runs were obtained from the European Genome Archive (EGA) under the accession number EGAD50000001710. The Tables of 5-mer counts detected for each RNA modification obtained from the analysis of published data [18] and the code used for this work can be found to the following GitHub page: https://github.com/mem3nto0/ONT_5mer_identifier.git

## Abbreviations

DRS: Direct RNA Sequencing
ONT: Oxford Nanopore Technology
