# Supplementary material for: Extracting candidate motif patterns for multiple RNA modifications using direct RNA sequencing
Source: BMC Bioinformatics. 2026 Oct 23;27:246. doi: 10.1186/s12859-026-06724-5 (PMC13700448; doi:10.1186/s12859-026-06724-5)
Supplement: Supplementary file 1 [file 12859_2026_6724_MOESM1_ESM.docx]

**Extracting candidate motif patterns for multiple RNA modifications using direct RNA sequencing**

**Supplementary Information**

**SI 1. Second population and database analysis**

**Supplementary Figure 1. Population analysis of low-frequency m⁶A motifs and database-derived RNA modification sites.** (a) Sequence logos of the second, low-frequency m⁶A population identified in HEK293T cells and human blood. (b–e) Exponential fitting and corresponding sequence logos obtained from modification sites reported in the m⁶A-Atlas v2.0, Ψ-Atlas, RMBase v3.0, and m⁵C-Atlas for m⁶A (b), pseudouridine (c), inosine (d), and m⁵C (e), respectively. In contrast to m⁶A, the database-derived sites for pseudouridine, inosine, and m⁵C do not reveal comparably distinct sequence preferences.

We first examined the second, low-frequency m⁶A population identified in HEK293T cells and human blood (Supplementary Figure 1a). In contrast to the high-frequency population, its sequence logos showed no clear enrichment of canonical DRACH motifs and displayed a less distinct sequence preference.

We subsequently applied the population-analysis framework to modification sites reported in the m⁵C-Atlas, m⁶A-Atlas v2.0, Ψ-Atlas, and RMBase v3.0 databases (1–4). The ranked distribution of database-derived m⁶A-associated 5-mers was fitted using a single exponential component (Supplementary Figure S1b), whereas the distributions obtained for Ψ, inosine, and m⁵C were fitted using two exponential components (Supplementary Figure S1c–e). The sequence logo generated from the database-derived m⁶A sites recapitulated the canonical DRACH motif. By contrast, the logos obtained for Ψ, inosine, and m⁵C showed no comparably distinct sequence preferences.

For Ψ, inosine, and m⁵C, the fitted amplitudes of the two exponential components were of similar magnitude (A₁ ≈ A₂; Supplementary Figure S1c–e), indicating that neither component clearly dominated the corresponding distributions. The databases for these modifications contain substantially fewer annotated sites than are currently available for m⁶A, and the reported sites were generated using heterogeneous experimental approaches. Their limited and uneven representation may therefore obscure high-frequency sequence preferences and reduce the statistical power of population-level motif analysis. Larger transcriptome-wide reference datasets containing independently validated modification sites will be required to determine whether reproducible motif families exist for these modifications.

The fitted parameters obtained from the modification calls in the HEK293T and peripheral blood datasets analysed in this study are provided below. The analysis included two HEK293T replicates (HEK293T_A and HEK293T_B) and three peripheral blood replicates (Blood_A, Blood_B, and Blood_C). Supplementary Tables SI1–SI4 report the fitted amplitudes (A₁ and A₂), decay constants (λ₁ and λ₂), and calculated cutoff indices for each RNA modification type and replicate. The fitting procedure and cutoff definition are described in the Methods section of the main text.

### **Supplementary Tables SI1–SI4**

**Supplementary Table SI1. Parameters obtained from double-exponential fitting of the ranked m⁶A 5-mer count distributions.** For each replicate, the table reports the fitted amplitudes A₁ and A₂, decay constants λ₁ and λ₂, and the calculated cutoff index.

| Samples | A_1_ | A_2_ | λ_1_ | λ_2_ | Cutoff index |
| --- | --- | --- | --- | --- | --- |
| Hek293T_A | 22801 | 3756 | 7.1 | 65.4 | 14 |
| Hek293T_B | 34026 | 5385 | 7.1 | 65.4 | 14 |
| Blood_A | 9000 | 842 | 4.2 | 34 | 8 |
| Blood_B | 22422 | 2555 | 4.2 | 34 | 8 |
| Blood_C | 15238 | 1237 | 4.2 | 34 | 8 |

| Samples | A_1_ | A_2_ | λ_1_ | λ_2_ | Cutoff index |
| --- | --- | --- | --- | --- | --- |
| Hek293T_A | 14036 | 2524 | 7.2 | 42.2 | 14 |
| Hek293T_B | 20920 | 3730 | 7.2 | 42.2 | 14 |
| Blood_A | 2109 | 678 | 3.5 | 14.6 | 7 |
| Blood_B | 5990 | 1818 | 3.5 | 14.6 | 7 |
| Blood_C | 4381 | 1017 | 3.5 | 14.6 | 7 |

**Supplementary Table SI2. Parameters obtained from double-exponential fitting of the ranked pseudouridine 5-mer count distributions.** For each replicate, the table reports the fitted amplitudes A₁ and A₂, decay constants λ₁ and λ₂, and the calculated cutoff index.

**Supplementary Table SI3. Parameters obtained from double-exponential fitting of the ranked inosine 5-mer count distributions.** For each replicate, the table reports the fitted amplitudes A₁ and A₂, decay constants λ₁ and λ₂, and the calculated cutoff index.

| Samples | A_1_ | A_2_ | λ_1_ | λ_2_ | Cutoff index |
| --- | --- | --- | --- | --- | --- |
| Hek293T_A | 3181 | 421 | 3.1 | 40 | 6 |
| Hek293T_B | 4629 | 621 | 3.1 | 40 | 6 |
| Blood_A | 1033 | 341 | 2.8 | 36.2 | 6 |
| Blood_B | 3030 | 969 | 2.8 | 36.2 | 6 |
| Blood_C | 2258 | 273 | 2.8 | 36.2 | 6 |

**Supplementary Table SI4. Parameters obtained from double-exponential fitting of the ranked m⁵C 5-mer count distributions.** For each replicate, the table reports the fitted amplitudes A₁ and A₂, decay constants λ₁ and λ₂, and the calculated cutoff index.

| Samples | A_1_ | A_2_ | λ_1_ | λ_2_ | Cutoff index |
| --- | --- | --- | --- | --- | --- |
| Hek293T_A | 4598 | 1179 | 2 | 48 | 4 |
| Hek293T_B | 6990 | 1918 | 2 | 48 | 4 |
| Blood_A | 1019 | 669 | 6.2 | 59.4 | 12 |
| Blood_B | 1892 | 1058 | 6.2 | 59.4 | 12 |
| Blood_C | 1060 | 653 | 6.2 | 59.4 | 12 |

**Supplementary Table SI5a. Dependence of the signal ratio on the cutoff parameter n.** Median signal ratios (S_R) were calculated for different values of n using Equation 2 of the main text. S_R describes the relative contribution of the high-frequency component compared with the low-frequency component.

| **n values** | **S_R_** |
| --- | --- |
| 1 | 2.23 |
| 1.5 | 1.45 |
| 2 | 0.99 |
| 2.5 | 0.66 |

| **m6A motifs** | | **Ψ motifs** | | **Inosine motifs** | | **m5C motifs** | |
| --- | --- | --- | --- | --- | --- | --- | --- |
| **Hek293T** | **Blood** | **Hek293T** | **Blood** | **Hek293T** | **Blood** | **Hek293T** | **Blood** |
| GGACA  (36461) | GGACU  (25567) | UUUUU  (21504) | CUUUG  (7582) | CAAGG  (4800) | UUAUU  (3673) | GACUG  (8893) | UGCAG  (2968) |
| GGACU  (35927) | GGACA  (19118) | CUUUG  (21218) | CUUUU  (5741) | GCAUU  (4510) | CUAUU  (3088) | GACAG  (4883) | CGCAG  (2806) |
| GAACU  (30573) | GAACU  (15097) | CUUUU  (20835) | CUUUA  (3553) | ACAUU  (3841) | CAAGG  (2448) | GACUU  (2529) | CGCGG  (2791) |
| AGACU  (27810) | AGACU  (15054) | CCUUU  (20745) | CUUUC  (2874) | UUAUU  (1841) | ACAUU  (2101) | GACUC  (2475) | GACUG  (2510) |
| GGACC  (26179) | GGACC  (12577) | CCUUC  (17578) | UUUUU  (2478) | CUAUU  (1830) | GCAUU  (1924) | - | GGCGG  (2235) |
| AAACU  (22534) | UGACU  (10800) | UUUUG  (13956) | UUUUG  (1457) | GCAGC  (1209) | GUAUU  (1391) | - | CACGG  (1942) |
| UGACU  (22041) | GGACU  (7706) | UUUUA  (13876) | UUUUA  (1254) | - | - | - | GGCAG  (1890) |
| GAACA  (20545) | AGACA  (5677) | CUUUA  (12880) | - | - | - | - | AACUG  (1654) |
| AGACA  (16505) | - | CUUUC  (11250) | - | - | - | - | CACAG  (1583) |
| AAACA  (11744) | - | UUUUC  (8458) | - | - | - | - | UGCGG  (1540) |
| GGACG  (11581) | - | CCUUG  (7106) | - | - | - | - | GACAG  (1386) |
| UGACA  (9693) | - | CUUGC  (5855) | - | - | - | - | CACCG  (1290) |
| AGACC  (8601) | - | CCUUA  (5712) | - | - | - | - | - |
| - | - | CAUUG  (5661) | - | - | - | - | - |

**Supplementary Table SI5b. High-frequency 5-mer motifs identified by population analysis.** The table lists the 5-mer sequences assigned to the high-frequency population for m⁶A, pseudouridine, inosine, and m⁵C in HEK293T cells and human blood. Motifs are ordered by decreasing abundance. The number of modification calls assigned to each 5-mer by the Dorado–Modkit workflow is shown in parentheses.

**Supplementary Table SI6. Numbers of RNA modification calls detected in each dataset.** For each replicate and modification type, the table reports the total number of sites passing the applied filtering criteria and the number assigned to the high-frequency population by the population analysis.

|  | **Replicate** | **m6A** | | **Ψ** | | **Inosine** | | **m5C** | |
| --- | --- | --- | --- | --- | --- | --- | --- | --- | --- |
|  |  | **Total calls** | High-frequency population | **Total calls** | High-frequency population | **Total calls** | High-frequency population | **Total calls** | High-frequency population |
| **Blood** | A | 66428 | 38951 | 17280 | 10153 | 15237 | 4270 | 46056 | 12950 |
|  | B | 181042 | 100444 | 47508 | 28237 | 43562 | 12363 | 74576 | 21983 |
|  | C | 106058 | 64545 | 30182 | 18914 | 16205 | 6883 | 45360 | 12990 |
| **Hek293T** | A | 407530 | 187920 | 207572 | 118176 | 26701 | 10945 | 65788 | 12476 |
|  | B | 593764 | 277625 | 308030 | 175746 | 39190 | 15974 | 106044 | 19449 |

**SI 2. Noise and reliability of the Dorado positions**

**Supplementary Figure 2. Dorado modification calls for synthetic oligonucleotides containing defined RNA modifications.** Dorado predictions are shown for ten oligonucleotides each containing m⁶A (a), pseudouridine (b), or inosine (c). Expected unmodified and modified positions are indicated by grey and black dashed lines, respectively.

To assess the accuracy of Dorado modification calling at independently established sites, we first analysed 30 previously published synthetic oligonucleotides containing defined RNA modifications: ten each for m⁶A, pseudouridine (Ψ), and inosine (5). The expected modified positions are indicated by black dashed lines in Supplementary Figure 2. Dorado detected the expected modification in 26 of the 30 oligonucleotides (86.7%). The four missed sites comprised one m⁶A- and three inosine-containing oligonucleotides. Thus, detection performance differed between modification types and sequence contexts.

Additional Dorado calls were observed at several positions surrounding the expected modification sites. However, more than half of these positions did not contain the canonical nucleotide corresponding to the modification under investigation and were therefore excluded by our motif-counting pipeline. For example, the two additional peaks observed for Psi_7 at positions 83 and 87 corresponded to cytosines rather than uridines in the reference sequence. These results demonstrate that the sequence and nucleotide filters applied by the pipeline remove a substantial proportion of incompatible calls, although they cannot eliminate false-positive calls occurring at the corresponding canonical nucleotide.

We next analysed published direct RNA sequencing data for human ribosomal RNA (6) and compared Dorado Ψ calls with 104 established Ψ sites identified by mass spectrometry (7). Dorado recovered 93 of the 104 known sites, corresponding to a recall of 89.4%. Conversely, 93 of the 115 positions called by Dorado matched established Ψ sites, corresponding to a precision of 80.9%. Dorado therefore recovered the majority of known rRNA Ψ sites but produced both false-negative and additional calls.

To investigate the possible influence of adjacent modifications, we compared regions containing densely clustered Ψ sites with regions containing more isolated sites (Supplementary Figure 3). Most established Ψ positions remained detectable in densely modified regions, but the corresponding modification frequencies estimated by Dorado were generally lower than the mass-spectrometry-derived reference values. This indicates that neighbouring modifications may affect quantitative frequency estimates without necessarily preventing positional detection. Because this analysis was restricted to Ψ in human rRNA, it does not exclude modification-dependent or sequence-dependent effects in other RNA contexts.

Collectively, the analyses of synthetic oligonucleotides and human rRNA show that Dorado recovers most independently established modification sites but does not provide error-free site-level predictions. These limitations are relevant when interpreting individual positions and modification frequencies. Nevertheless, the recovery of the majority of expected sites supports the use of the resulting calls for population-level motif analysis, provided that the inferred motif patterns are interpreted as candidates requiring independent biological validation.

**References**

**Supplementary Figure 3. Comparison of Dorado Ψ predictions with established modification sites in human ribosomal RNA.** Dorado-derived modification frequencies are shown in orange and mass-spectrometry-derived reference values in blue. Examples include regions containing densely clustered Ψ sites (a, b) and more isolated Ψ sites (c, d). In densely modified regions, most established sites remained detectable, although their modification frequencies tended to be underestimated.

1.	Xuan, J. *et al.* RMBase v3.0: decode the landscape, mechanisms and functions of RNA modifications. *Nucleic Acids Res.* **52**, D273–D284 (2024).

2.	Liang, Z. *et al.* m6A-Atlas v2.0: updated resources for unraveling the N6-methyladenosine (m6A) epitranscriptome among multiple species. *Nucleic Acids Res.* **52**, D194–D202 (2024).

3.	Wang, X. *et al.* Ψ-Atlas: an integrated atlas for pseudouridine epitranscriptome. 2025.06.06.656536 Preprint at https://doi.org/10.1101/2025.06.06.656536 (2025).

4.	Ma, J. *et al.* m5C-Atlas: a comprehensive database for decoding and annotating the 5-methylcytosine (m5C) epitranscriptome. *Nucleic Acids Res.* **50**, D196–D203 (2022).

5.	Alagna, N. *et al.* ModiDeC: a multi-RNA modification classifier for direct nanopore sequencing. *Nucleic Acids Res.* **53**, gkaf673 (2025).

6.	Pastore, S. *et al.* Mapping human pre-rRNA processing and modification at single nucleotide resolution using long read nanopore sequencing. *Nat. Commun.* **17**, 4658 (2026).

7.	Taoka, M. *et al.* Landscape of the complete RNA chemical modifications in the human 80S ribosome. *Nucleic Acids Res.* **46**, 9289–9298 (2018).
